# Influence of Irradiated Peripheral Blood Mononuclear Cells on Both *Ex Vivo* Proliferation of Human Natural Killer Cells and Change in Cellular Property

**DOI:** 10.3389/fimmu.2017.00854

**Published:** 2017-07-24

**Authors:** María Delso-Vallejo, Jutta Kollet, Ulrike Koehl, Volker Huppert

**Affiliations:** ^1^Miltenyi Biotec GmbH, Bergisch-Gladbach, Germany; ^2^Hannover Medical School, Institute for Cellular Therapeutics, IFB-Tx, Hannover, Germany

**Keywords:** natural killer cells, *ex vivo* expansion, immunotherapy, HLA-DR, RANKL, B7-H3

## Abstract

Clinical studies with adoptive immunotherapy using allogeneic natural killer (NK) cells showed feasibility, but also limitation regarding the transfused absolute cell numbers. First promising results with peripheral blood mononuclear cells (PBMCs) as feeder cells to improve the final cell number need further optimization and investigation of the unknown controlling mechanism in the cross-talk to NK cells. We investigated the influence of irradiated autologous PBMCs to boost NK cell proliferation in the presence of OKT3 and IL-2. Our findings demonstrate a requirement for receptor–ligand interactions between feeders and NK cells to produce soluble factors that can sustain NK cell proliferation. Thus, both physical contact between feeder and NK cells, and soluble factors produced in consequence, are required to fully enhance NK cell *ex vivo* proliferation. This occurred with an indispensable role of the cross-talk between T cells, monocytes, and NK cells, while B cells had no further influence in supporting NK cell proliferation under these co-culture conditions. Moreover, gene expression analysis of highly proliferating and non-proliferating NK cells revealed important phenotypic changes on 5-day cultured NK cells. Actively proliferating NK cells have reduced Siglec-7 and -9 expression compared with non-proliferating and resting NK cells (day 0), independently of the presence of feeder cells. Interestingly, proliferating NK cells cultured with feeder cells contained increased frequencies of cells expressing RANKL, B7-H3, and HLA class II molecules, particularly HLA-DR, compared with resting NK cells or expanded with IL-2 only. A subset of HLA-DR expressing NK cells, co-expressing RANKL, and B7-H3 corresponded to the most proliferative population under the established co-culture conditions. Our results highlight the importance of the crosstalk between T cells, monocytes, and NK cells in autologous feeder cell-based *ex vivo* NK cell expansion protocols, and reveal the appearance of a highly proliferative subpopulation of NK cells (HLA-DR^+^RANKL^+^B7-H3^+^) with promising characteristics to extend the therapeutic potential of NK cells.

## Introduction

Among the different approaches of immunotherapy to treat cancer, natural killer (NK) cells are very promising cell types with impressive outcomes in clinical studies. NK cells are innate lymphoid cells (1). They are characterized by their potent cytotoxic responses against virus-infected and malignantly transformed cells, without the need of prior immune sensitization, and in a major histocompatibility complex-unrestricted manner (2, 3). In addition, NK cells produce cytokines such as TNF-α and IFN-γ, which enhance immune responses, and engage in reciprocal interactions with other immune cells that contribute to different immune responses including anti-tumor effects (4).

To date, allogeneic NK cells for adoptive immunotherapy have already entered clinical studies successfully for both applications, post stem cell transplantation (5, 6), and in non-transplant settings to treat cancer patients (7–9). However, manufacturing of NK cells directly isolated from apheresis products can result in varying quantity (10, 11) and yield not always sufficient amounts to carry out multiple applications (12–14). An increase in the number of functional NK cells by *ex vivo* expansion methods is therefore of high interest and has recently been summarized (13).

Natural killer cells require multiple signals for their *ex vivo* survival, proliferation, and activation, involving soluble factors and the necessity of physical interactions with other cells. All of these components can be conveniently supplied by feeder cells (14, 15). Different types of feeder cells have been tested for their potential in supporting NK cell *ex vivo* expansion from both, autologous or allogeneic origin. Typically, they are irradiated prior to use and supplemented with survival and activating factors such as the cytokines IL-2 and IL-15 and/or the anti-CD3 monoclonal antibody (mAb) OKT3. Several approaches using autologous peripheral blood mononuclear cells (PBMCs) as feeder cells have demonstrated their utility to generate sufficient NK cell numbers for clinical applications (16–19). In terms of clinical manufacturing, autologous PBMCs are the preferable choice to avoid safety issues that allogeneic feeder cells may rise. Despite these advantages, little is known about the positive effect of autologous feeder cells on NK cell proliferation and activation. A beneficial role of monocytes in promoting NK cell *ex vivo* proliferation has been proposed (20). However, the underlying cellular and molecular changes that NK cells undergo during active proliferation yet need to be unraveled.

In this study, we established a co-culture system with autologous PBMCs to examine which components have a significant influence concerning the enhancement of NK cell proliferation. We further characterized the cellular and molecular changes occurring in actively proliferating NK cells. Our data provide a better understanding of mechanisms influencing and modulating *ex vivo* NK cell proliferation and might be the base to improve harmonized manufacturing protocols for future clinical NK cell studies.

## Materials and Methods

### Cells and Cell Lines

Buffy coats from healthy donors (Klinikum Dortmund) were used for PBMC isolation. Daudi JP cells were a kind gift of Dr. R. Seggewiss-Bernhardt, University Hospital of Wuerzburg, who obtained them from Prof. P. Fisch, University of Freiburg (21), and K562 cells were obtained from the German Collection of Microorganisms and Cell Cultures (DSMZ). Both cell lines were maintained in complete RPMI medium, RPMI 1640 (Biowest) supplemented with 10% fetal bovine serum (Biochrom), and 2 mmol/L L-glutamine (PAA), in a humidified atmosphere with 5% CO_2_ at 37°C.

### NK Cell Isolation and Co-Culture with Autologous PBMCs

Peripheral blood mononuclear cells were isolated from buffy coats by density gradient centrifugation using Pancoll (PAN-Biotec). To obtain different feeder cell fractions, PBMCs were depleted first of CD56^+^ cells by MACS sort using anti-CD56 microbeads (Miltenyi Biotec), and when indicated they were further depleted of CD19^+^ and CD3^+^ or CD14^+^ cells using the corresponding microbeads (Miltenyi Biotec). The differently depleted PBMCs fractions were X-ray irradiated (20 Gy) (RS 2000 Biological Research Irradiator, Radsource) and used as autologous feeder cells for co-culture with NK cells within 1–1.5 h post-irradiation. The irradiated autologous CD56-depleted PBMCs (“IAPs”) were used as the major feeder cells. NK cells were purified from PBMCs using the human NK cell isolation kit (Miltenyi Biotec), and expanded in 24-well plates, either in co-culture with autologous feeder cells at a 20:1 feeder-NK cell ratio, based on protocol from Ahn et al. (16), or without feeder cells, in complete culture medium; TexMACS medium (Miltenyi Biotec) supplemented with 5% human AB serum (Life Technologies) and 1,000 U/mL of Proleukin S (rhIL-2) (Novartis). Co-cultures were additionally supplemented with 10 ng/mL of anti-CD3 mAb (functional grade OKT3, Miltenyi Biotec). When indicated, NK cells were labeled prior cultivation with CellTrace™ Violet Cell Proliferation Kit [cell trace violet (“CTV”) dye] (Life Technologies). Initial total cell densities of cultures were 1 × 10^6^ cells/mL. Culture plates were incubated in a humidified atmosphere with 5% CO_2_ at 37°C. To determine NK cell fold expansions, NK cell densities were checked at different indicated time points by volumetric counting and detection of viable CD3^−^CD56^+^ cells using MACSQuant Analyzer 10 (Miltenyi Biotec). For 12-day expansions, NK cells were harvested from 24-well plates on day 7 and transferred to 25 or 75 T-flasks with replenishment of fresh medium without OKT3. Fresh medium was additionally replenished on day 9 or 10.

### Co-Cultures in Transwell^®^ Plates

Purified NK cells were cultured for up to 5 days with or without IAPs in 12-well polystyrene plates equipped with Transwell^®^ inserts (Costar). The insert system consisted of a 500 µL upper well (12 mm diameter), separated from the bottom well (1.5 mL) by a 0.4 µm microporous tissue culture-treated polycarbonated membrane. All cells were resuspended in complete culture medium and IAPs suspensions were supplemented with OKT3 as described. Four different culture conditions were established, all of them containing 1 mL of NK cells (5 × 10^5^ cells/mL) at the bottom wells. In two conditions, either 1 mL of complete medium or 1 mL of IAP suspension were added to the NK cells on the bottom wells, and upper wells were left empty. In two other conditions, either 1 mL of IAP suspension (10 × 10^6^ cells/mL) was inoculated through the insert, or a combination of 0.5 mL of IAPs (10 × 10^6^ cells/mL) and 0.5 mL of purified NK cells (5 × 10^5^ cells/mL) was inoculated, remaining the inoculated cells in both situations on the upper well of the insert. Transwell plates were incubated in a humidified atmosphere with 5% CO_2_ at 37°C.

### Flow Cytometry

Natural killer cell frequencies were determined using the following panel of mAbs from Miltenyi Biotec: CD45-VioGreen (5B1), CD3-FITC (BW264/56), BDCA1-PE (AD5-8E7), CD14-PE (TÜK4), CD19-PEVio770 (LT19), CD16-APC (VEP13), and CD56-APCVio770 (REA196). In experiments with feeder cell fractions containing additional depletions of CD19^+^ and CD3^+^ or CD14^+^ cells, the mAb BDCA-2-PE (AC144) was included instead of CD14-PE, and CD16-APC replaced by CD14-APC. The human FcR Blocking Reagent from Miltenyi was also used to block unspecific antibody binding to Fc receptors on CD14^+^ enriched feeder cell fractions. Propidium iodide (Miltenyi Biotec) was used at a final concentration of 1 µg/mL to exclude dead cells from the analysis.

To study the phenotypic differences between resting, proliferating, and non-proliferating NK cells, cells were labeled prior cultivation with or without IAPs, with the cell trace proliferation dye eFluor^®^670 (eBioscience), in order to differentiate proliferating and non-proliferating cells. Different candidate molecules were analyzed using several antibody panels designed based on previous work (22). These panels shared a backbone of mAbs: CD45-VioGreen, CD3-VioBlue, TCRγδ-VioBlue (11F2), CD14-VioBlue, CD19-VioBlue, and CD56-APCVio770, and SYTOX^®^ Blue (Life Technologies) was used to exclude dead cells from the analysis. The backbone was combined with groups of the following mAbs (Miltenyi Biotec unless otherwise indicated): KLRB1-FITC (191B8), CTLA-4-PE (BNI3), Siglec-9-PEVio770 (REA492), Siglec-7-PerCP700 (REA214), LILRB1-FITC (GHI/75), KLRG1-PE (REA261), CD16-PerCP700 (VEP13), RANKL-PE (DN254), B7-H3-PEVio770 (FM276), NKp44-PEVio770 (2.29), NKp80-FITC (4A4.D10), 4-1BB-PE (4b4-1), 2B4-PEVio770 (REA112), NKG2D-PerCPCy5.5 (1D11) (Biolegend), HLA-DP/DQ/DR-FITC (REA332), ALCAM-PEVio770 (REA442), HLA-A/B/C-PerCPVio700 (REA230), and HLA-DR-FITC (AC122). The corresponding mouse immunoglobulin (Ig) G1, IgG2A, IgG2B, IgM, or REAs conjugated with the respective dyes were used as isotype controls. Cells were acquired using MACSQuant Analyzer 10 and analyzed using MACSQuantify 2.8 software (Miltenyi Biotec).

### Cytokine Detection

Supernatants from co-cultures of NK cells with IAPs further depleted of CD19^+^, and CD3^+^ or CD14^+^ cells were collected after 5 days and cytokine production was detected using the flow cytometry bead-based array MACSPlex Cytokine 12 kit, human according to manufacturer’s instructions (Miltenyi Biotec). The MACSPlex Cytokine 12 kit allows for the detection of human GM-CSF, IFN-α, IFN-γ, IL-2, IL-4, IL-5, IL-6, IL-9, IL-10, IL-12p70, IL-17A, and TNF-α. Samples were acquired using a MACSQuant Analyzer 10 and analyzed using the Express Mode option of the MACSQuantify 2.8 software (Miltenyi Biotec).

### Cytotoxicity Assays

Target cell killing of K562 and Daudi JP cell lines was analyzed using a flow cytometry-based assay (23). Briefly, target cells were labeled with CTV dye and seeded in 96 well U-bottom plates at a cell density of 2 × 10^5^ cells/mL, and incubated alone, or with expanded NK cells (2 × 10^6^ cells/mL) at different effector-to-target (E:T) ratios, for 4 h in a humidified atmosphere with 5% CO_2_ and 37°C. Antibody-dependent cellular cytotoxicity (ADCC) of expanded NK cells was further analyzed against the CD20^+^ Daudi cells, by adding 5 µg/mL of the anti-CD20 mAb rituximab (Hoffman-La Roche). After 4 h incubation, plates were transferred to 4°C for at least 30 min to stop cell killing before quantifying viable CTV-positive target cells using the MACSQuant Analyzer 10. The frequency of killed target cells was calculated by the difference between the number of viable target cells in samples with effector NK cells and samples with targets cells containing no effector cells. Representative flow cytometry data are included in Figure S1 in Supplementary Material.

### Preparation of NK Cells for Sorting

Natural killer cells from five different donors were labeled with the cell trace proliferation dye eFluor^®^670 (eBioscience) and co-cultured for 5 days with IAPs. On day 5, cells were harvested and proliferating and non-proliferating NK cells were sorted according to the brightness of the cell trace dye in a FACSAria III cytometer (BD Bioscience, cell sorting facility of the Center for Molecular Medicine of Cologne, Cologne, Germany). 7-AAD (BD Pharmigen™) was used to exclude dead cells. To ease the sorting process, prior to sort, samples were enriched on NK cell content using the human NK cell isolation kit from Miltenyi. Sorted fractions containing the proliferating and non-proliferating (≈0.8 × 10^5^ cell in each fraction) were lysed in RA1 buffer (Macherey-Nagel) for total RNA isolation, and stored at −20°C until use. For comparison, resting NK cells (1 × 10^6^ cells) were lysed and stored under identical conditions.

### RNA Microarrays

Total RNA from sorted samples was isolated using the NucleoSpin^®^ RNA kit (Macherey-Nagel), amplified and labeled using the Agilent Low Input Quick Amp Labeling Kit (Agilent Technologies), prior to hybridization to Agilent Whole Human Genome Oligo Microarrays 8 × 60K V2 chips (Agilent Technologies) according to manufacturer instructions. A detailed description of microarray processing can be found in Supplementary Material.

### Pre-Processing of Microarray Data

Raw intensity data from feature extraction output files (FES 10.7.3.1, Agilent Technologies) were analyzed using the Rosetta Resolver^®^ software (Rosetta Biosoftware). The following calculations were performed with software packages within R/Bioconductor (24, 25). Intensity values were corrected with background subtraction and normalized by quantile normalization (26). Reliable signal intensities were considered significant when *p* ≤ 0.01, using the Rosetta error model (27). Subsequent statistical analysis was performed on normalized Log2-transformed intensity values. The data set can be found at the NCBI GEO public database with the accession number GSE92512.

### Statistical Analysis

Conventional statistics including parametric un-paired Student’s *t*-test and one-way ANOVA with Tukey’s *post hoc* test, or the non-parametric Wilcoxon test and Kruskal–Wallis test with *post hoc* Dunn’s test were performed with Graph Pad Prism 7 software (GraphPad). All statistical analysis were two-sided and *p* < 0.05 considered statistically significant, and indicated as *p < 0.05, **p < 0.01, and ***p < 0.001. Only statistical differences are shown.

Description of the statistical analysis of the microarray data can be found in Supplementary Material.

## Results

### Enhancement of *Ex Vivo* NK Cell Proliferation and Preservation of Function Using Irradiated Autologous CD56-Depleted PBMCs

**Figure 1:**
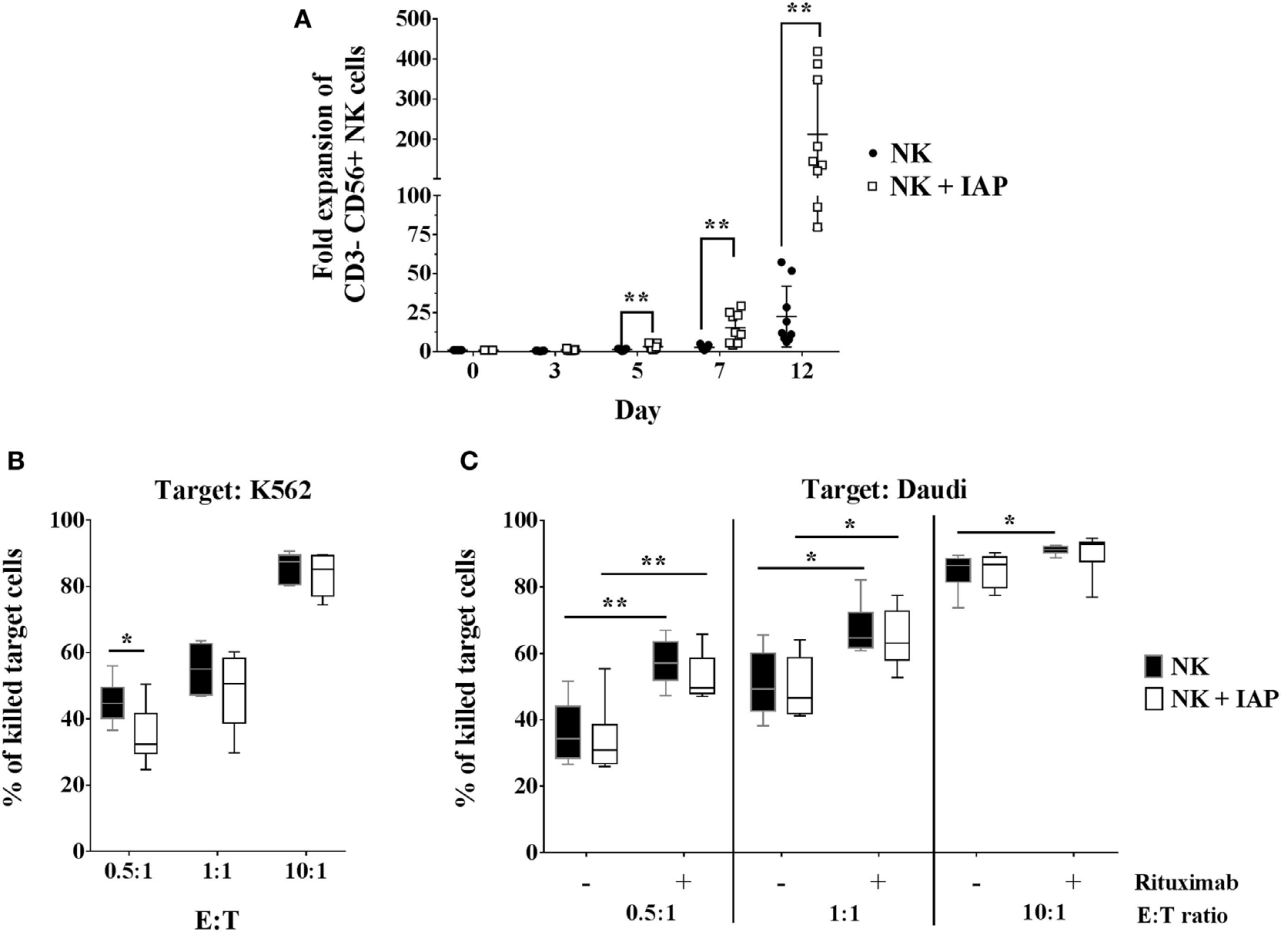
Natural killer (NK) cell expansion using autologous CD56-depleted peripheral blood mononuclear cells (PBMCs) and cytotoxic responses compared with standard culture with rhIL-2 only. **(A)** Proliferation kinetics of NK cells in expansion with irradiated autologous CD56-depleted PBMCs (IAPs) or NK cells from the same donors but cultured with rhIL-2 only were determined by flow cytometry (*n* = 9). A separate set of samples was cultured using both methods to compare expansions up to 12 days (also *n* = 9). Fold expansion of cultured relative to resting CD56^+^ NK cells are shown for each group per donor including mean ± SD. The non-parametric Wilcoxon test was used for statistical analysis. Natural cytotoxicity of 12-day expanded NK cells with and without IAPs was assessed against K562 **(B)** and Daudi cell lines, including antibody-dependent cellular cytotoxicity triggered by rituximab **(C)**. Frequencies of killed target cells were determined by flow cytometry and shown as mean, minimum to maximum, and SD. Statistical analysis was performed using un-paired Student’s *t*-test. Figure S1 in Supplementary Material shows representative raw flow cytometry data of cytotoxicity assays.

We first established an NK cell expansion method using irradiated autologous CD56-depleted PBMCs (IAPs) as feeder cells to achieve high expansion rates compared with culture with rhIL-2 only. We compared the proliferation kinetics over 7 days of NK cells cultured with IAPs or with rhIL-2 only. NK cells cultured with IAPs showed significantly higher cell counts visible already at day 5 (mean fold expansion: 3.2, range: 1.4–5.7) compared with cultivation with rhIL-2 only (mean fold expansion: 1.3, range: 0.4–1.9). This differential NK cell expansion rate between the two cultivation methods became much more pronounced after prolonged cultivation, as observed in a set of NK cell expansions over 12 days. Here, cells cultured with IAPs reached particularly high cell numbers (mean fold expansion: 212, range: 80–419) compared with rhIL-2 cultivation only (mean fold expansion: 22.5, range: 6.2–57.3) (Figure 1A). The killing capacity of the differently expanded NK cells was tested against two different leukemia tumor cell lines, the K562 cell line to analyze natural cytotoxicity, and the CD20^+^ Daudi cells to also determine ADCC after opsonization of these cells with anti-CD20 mAb rituximab. The killing capacity of the differently expanded cells was preserved in all tested settings. NK cells displayed comparable natural killing rates toward K562 and Daudi targets (Figures 1B,C; Figure S1 in Supplementary Material), as well as comparable ADCC responses upon rituximab opsonization of Daudi cells. *In vitro* ADCC responses can be monitored best at low E:T ratios, where read-out of natural killing activity is lower. Independent of the expansion method, NK cells showed a clearer ADCC effect of rituximab-coated target cells at low E:T ratios (Figure 1C; Figure S1 in Supplementary Material). Taken together, co-culture with IAPs significantly enhanced *ex vivo* NK cell proliferation preserving their cytotoxic capacity.

### Cross-Talk of NK Cells with Both T Cells and Monocytes Is Necessary to Produce Soluble Factors that Enhance *Ex Vivo* NK Cell Proliferation

**Figure 2:**
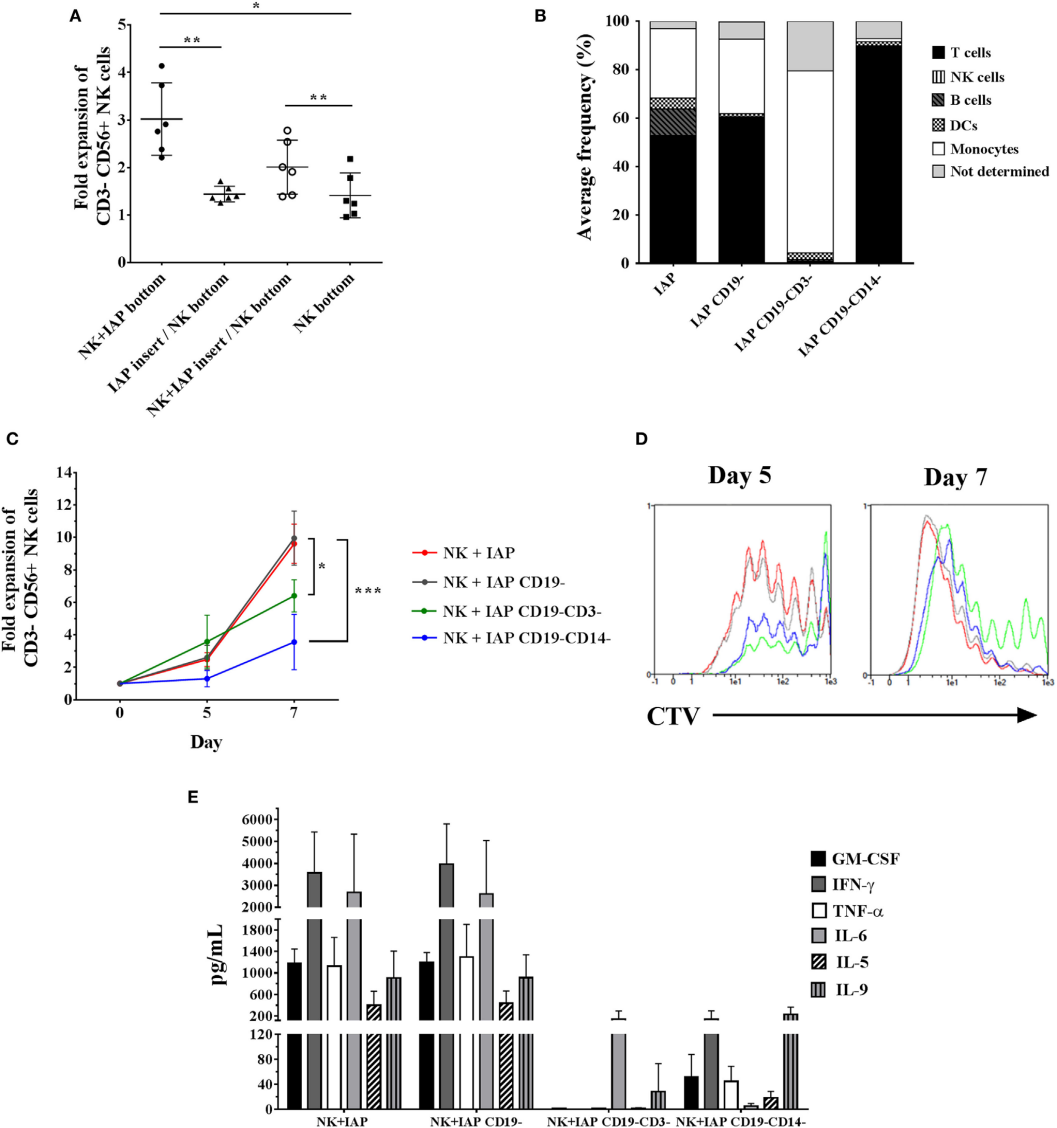
Influence of cell-to-cell interactions from different IAP feeder cell fractions on natural killer (NK) cell expansion and cytokine production upon co-culture. **(A)** NK cell counts were assessed on day 5 of culture in Transwell^®^ plates either alone (NK bottom) or co-cultured with IAPs. For co-cultures, NK cells were either kept in close contact with IAPs (NK + IAP bottom), or IAPs were kept separated from NK cells through an insert with microporous membrane in two conditions: IAPs alone in the insert (IAP insert/NK bottom), or with NK cells (NK + IAP insert/NK bottom). Mean values and SD of NK cell fold expansions from six different donors are depicted. Statistical analysis was performed using one-way ANOVA with Tukey’s *post hoc* test and Greenhouse–Geisser adjustment for unequal variances. **(B)** Composition of different IAP fractions analyzed by flow cytometry on day 0 after differential depletions of CD19^+^ (IAP CD19^−^) and additional CD3^+^ (IAP CD19^−^CD3^−^) or CD14^+^ (IAP CD19^−^CD14^−^) cells. Average frequencies shown are of four donors. **(C)** NK cell fold expansions analyzed on days 5 and 7 of co-culture with differently depleted IAP fractions (*n* = 4) were determined by flow cytometry. Statistical analysis was performed using one-way ANOVA with Tukey’s *post hoc* test. **(D)** Cell trace violet dilution of NK cells expanded with the differently depleted IAP fractions was measured by flow cytometry on days 5 and 7 of co-culture. A representative result of four different donors is shown. **(E)** Cytokine expression levels in supernatants after 5 days of NK cell co-cultures with the differently depleted IAP fractions were determined by cytometric bead array (*n* = 4).

Next, we sought to determine whether the enhancement of NK cell *ex vivo* proliferation with our established protocol is a consequence of direct cell-to-cell interactions between NK and feeder cells, or of exposure to soluble factors released by feeder cells. To address this question, we cultured NK cells for 5 days at the bottom of Transwell^®^ plates, alone or with IAPs either in close contact or separated through a permeable membrane, allowing only traffic of soluble factors. NK cell fold expansions were significantly decreased when IAPs were separated through the membrane, reaching similar fold expansions as NK cells cultured alone. On the other hand, the addition of NK cells to IAPs separated by the membrane, rescued the expansion of the NK cells at the bottom of the Transwells (Figure 2A). These data indicate that cell-to-cell interactions between IAPs and NK cells are necessary to produce soluble factors beneficial for NK cell proliferation.

IAPs are composed of different immune cells, mainly T cells, B cells, monocytes, and dendritic cells. In this respect, we speculated whether any of these cells could have a predominant role in stimulating NK cell proliferation. NK cell fold expansions were compared after co-culture with a standard IAP feeder cell fraction and three other fractions that were depleted from CD19^+^ cells (B cells and a small subset of dendritic cells), or additionally further depleted from CD3^+^ (T cells) or CD14^+^ cells (monocytes), respectively (Figure 2B). Depletion of CD19^+^ cells from the standard IAP fraction, had no effect on NK cell proliferation, whereas further depletion of T cells or monocytes significantly reduced NK cell expansion, with a more dramatic effect upon the absence of monocytes (Figure 2C). Differences in NK cell fold expansions correlated with differences in number of dividing cells as observed with dilution of CTV dye labeling (Figure 2D).

We hypothesized that the differences in stimulating NK cell proliferation could be mirrored in the cytokine composition released by the different feeder cell fractions. GM-CSF, IFN-γ, IL-6, TNF-α, IL-5, and IL-9 could be detected at high levels (>100 pg/mL) and were produced at a similar extent in co-cultures of NK cells with IAPs or with CD19-depleted IAPs. A remarkable reduction in cytokine production was observed in co-cultures with further depletion of T cells or monocytes, respectively (Figure 2E). This, indeed, supported the differences observed in NK cell proliferation kinetics due to the different cellular composition of the feeder cell fractions. The presence of NK cells in co-culture with the different feeder cell fractions influenced the cytokine production, as observed when feeders were maintained in culture without NK cells (Figure S2 in Supplementary Material). Low detectable levels (<100 pg/mL) of IL-4, IL-10, IL-17A, IFN-α, and IL-12 were also found in all co-culture conditions, without prominent changes upon differential cell subsets depletions from IAPs (data not shown). IL-2 was detected at levels exceeding the maximum standard value (>10.000 pg/mL) in any co-culture condition, as a result of its supplementation at the beginning of the culture (data not shown); which was sufficient to support NK cell growth independently of the presence or absence of T cells and monocytes. Taken together, our data indicate that the cross-talk between NK cells, T cells, and monocytes, is crucial to enhance *ex vivo* NK cell proliferation when using IAPs, not only due to occurrence of receptor–ligand interactions among these cells but also possibly due to the production of soluble factors as consequence of this cross-talk.

### Transcriptional Analysis of Expanded NK Cells Co-Cultured with IAPs Helps to Unravel Characteristics of Highly Proliferating NK Cells

**Figure 3:**
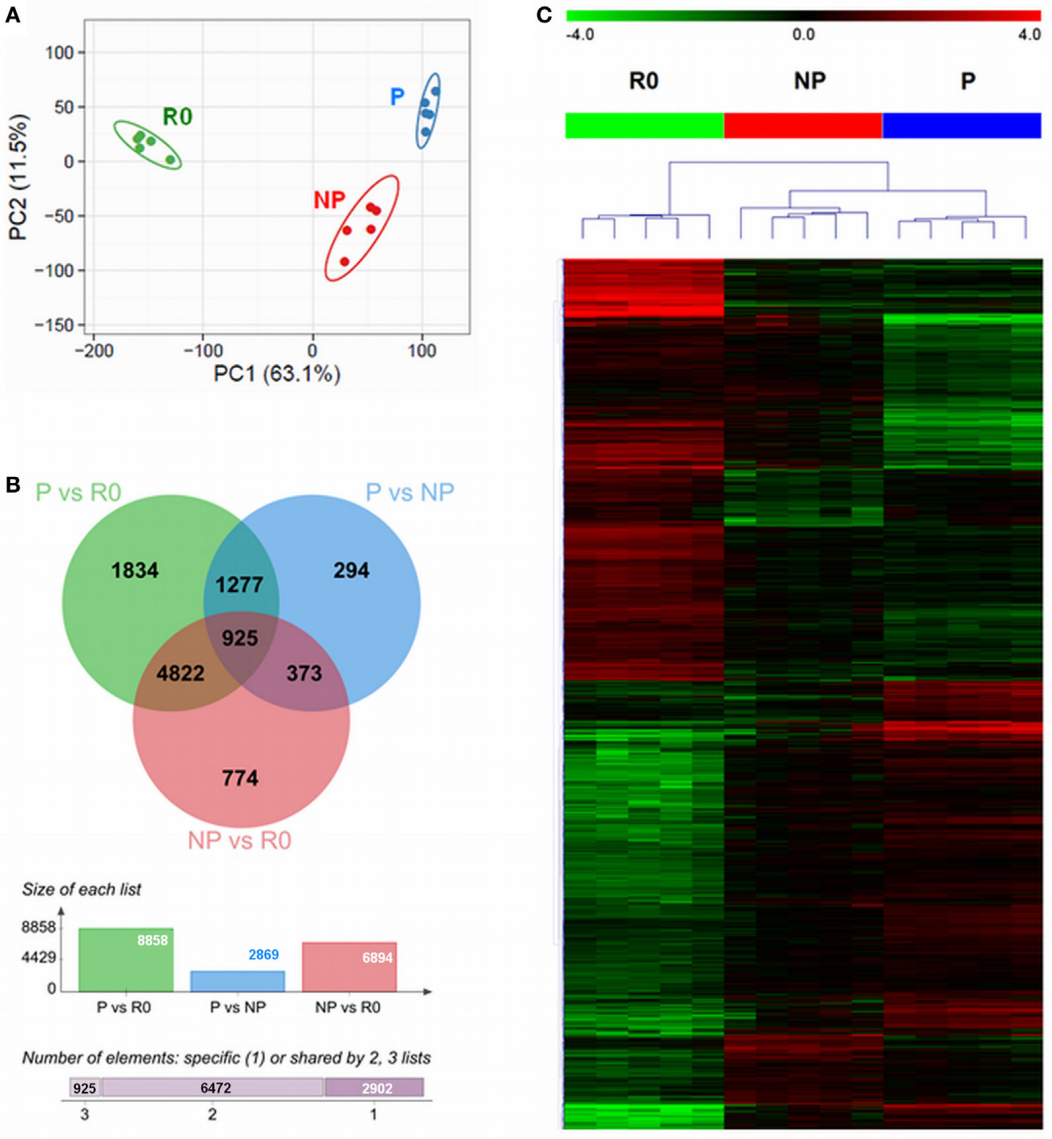
Differential transcriptome signatures between resting, proliferating, and non-proliferating natural killer (NK) cells expanded with IAPs. **(A)** Unsupervised principal component analysis of the transcriptomes of all samples (*n* = 5) for resting (R0); proliferating (P), and non-proliferating (NP) NK cells, displays the samples in a scatterplot of principal component 1 and principal component 2. The prediction ellipses are indicative of that with a 95% probability a new observation from the same group will fall inside the ellipse. **(B)** Venn diagram (top) displaying the overlap between the numbers of differentially expressed transcripts of each pairwise comparison, PvsR0, PvsNP, and NPvsR0. The columns diagram (middle) indicate the total number of differentially expressed reporters per pairwise comparison, and the horizontal bar (bottom) summarizes the number of transcripts specific or shared between two or three groups. **(C)** Heat map of all differentially expressed transcripts after hierarchical clustering (Euclidean distance, complete linkage method). Fold-change differences (row-wise centered to the median) are displayed within color saturation limits −4 (green) to +4 (red).

The multitude of signals occurring during the co-culture of NK cells with IAPs helped to boost the *ex vivo* proliferation of NK cells. However, at early time points during culture (day 5) not all NK cells are actively proliferating, but some remain quiescent. We aimed at identifying molecules or pathways responsible for the boost of *ex vivo* NK cell proliferation under the co-culture conditions. For this purpose, we performed a whole genome microarray analysis, with five different donors, to study the transcriptome of resting (R0) NK cells, isolated on day 0, and compare it to proliferating (P), and non-proliferating (NP) NK cells co-cultured for 5 days with IAPs (Figure S3 in Supplementary Material). Principal components analysis (PCA) of the unfiltered transcriptomes separated the samples in three groups corresponding to resting, proliferating, and non-proliferating NK cells, respectively (Figure 3A). The expression differences between R0 and 5-day cultured samples appear to contribute most to the variation (principal component 1, 63.1%), while differences between P and NP can be ascribed to the second principal component (principal component 2, 11.5%). A total of 10,299 transcripts were differentially expressed among the three groups, only 2,902 of these transcripts were unique candidates (Figure 3B). Hierarchical clustering of the differentially expressed transcripts confirmed the PCA results of the unfiltered data by the formation of two main clusters according to the samples of resting and 5-day expanded NK cells, the latter separated in sub-clusters corresponding to P and NP (Figure 3C). A functional annotation analysis of all differentially expressed transcripts revealed to which biological processes and pathways the transcripts were related to. In addition to the anticipated associations with IL-2 pathway, the great majority of the identified transcripts were associated with nucleotide metabolism and categories principally related to regulation of cell proliferation and activation (Figure S4 in Supplementary Material). This demonstrates that the sorted fractions indeed represented proliferating and non-proliferating cells.

**Figure 4:**
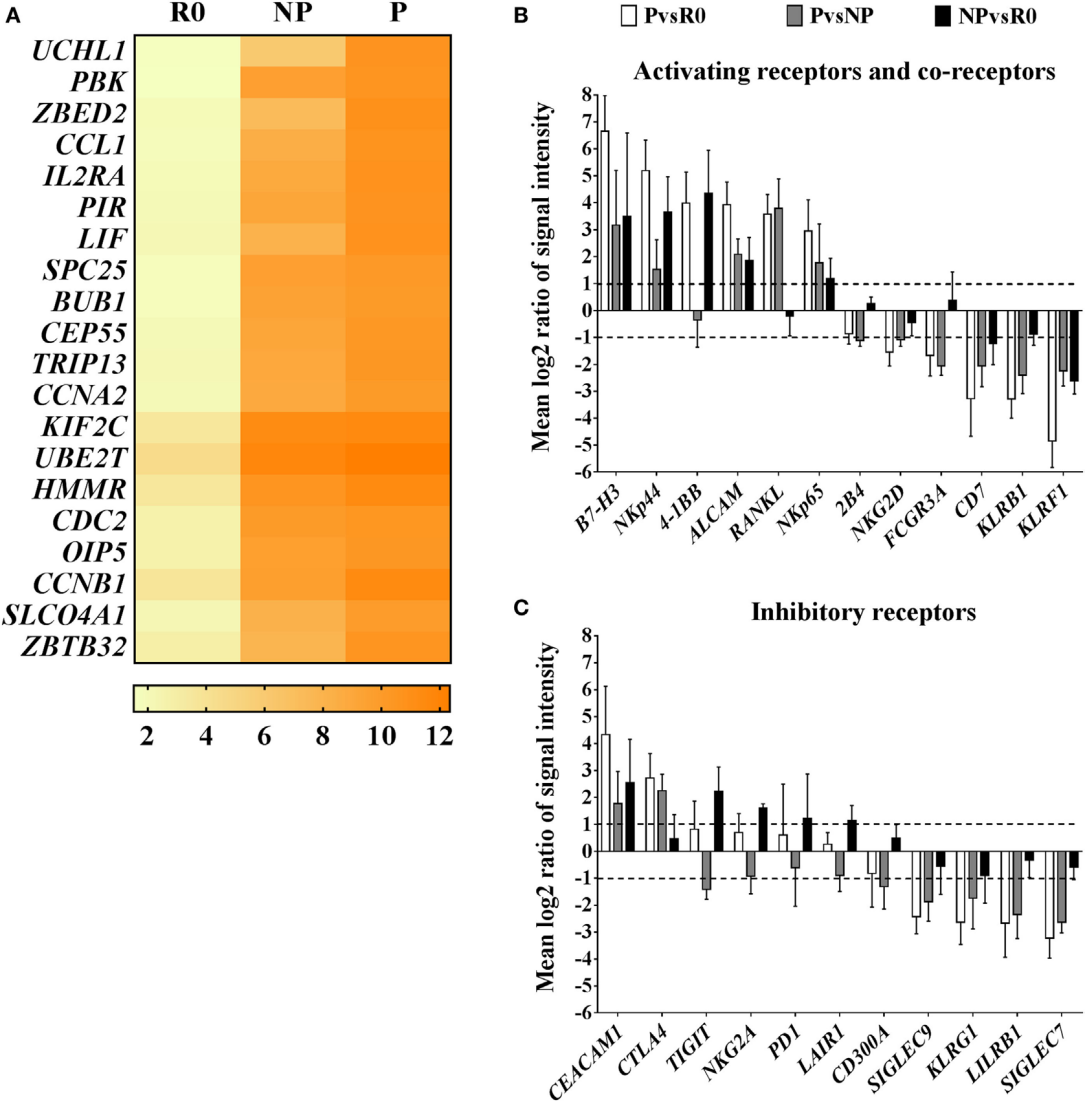
Transcriptional characteristics of proliferating natural killer (NK) cells compared with their non-proliferating counterparts and resting NK cells. **(A)** Top 20 transcripts with highest expression in proliferating NK cells (P) compared with resting cells (R0). Values in the heat map correspond to mean log2 intensities per group (*n* = 5) for each transcript. **(B)** Pairwise analysis (PvsR0, PvsNP, NPvsR0) of differentially expressed transcripts corresponding to activating receptors and co-receptors, or inhibitory receptors **(C)**. Pairwise comparisons are reported as mean values of log2 ratios of signal intensities with SD (*n* = 5). Only transcripts with differential expression levels (mean log2 ratios ≥1 or ≤−1, equivalent to ≥2 or ≤−2-fold difference) in at least one of the pair wise group comparisons are displayed.

In this regard, among the top 20 most highly expressed transcripts in proliferating NK cells compared with resting cells, were several positive regulators of the cell cycle and mitosis, such as cyclin A2 (*CCNA2*) and centrosomal protein of 55 kDa (*CEP55*), or the zinc finger proteins *ZBED2* and *ZBTB32* (Figure 4A). Zinc finger proteins are structurally a heterogeneous group of molecules which regulate gene expression by binding to DNA and RNA (28). Particularly, Tramtrack bric á brac zinc finger proteins (BTB-ZF) have important roles in controlling development and functional activity of lymphocytes (29, 30). Our data show that in proliferating NK cells, *ZBED2* was the zinc finger protein with highest transcript levels, and the transcription factor *ZBTB32* was the family member of BTB-ZF proteins with highest transcript levels (Figure S5A in Supplementary Material). These results point to a potential role of zinc finger proteins in modulating human *ex vivo* NK cell proliferation.

Natural killer cell receptors and co-receptors play key roles in controlling NK cell activation. We additionally screened for the most well-known activating and inhibitory receptors and co-receptors differentially expressed among the groups. In general, transcript levels of activating receptors were increased in proliferating cells, meanwhile transcripts of many inhibitory receptors, were decreased (Figures 4B,C). Interestingly, among activating receptors and co-receptors, we found that proliferating NK cells compared with resting or non-proliferating contained increased transcript levels of receptor activator of the nuclear factor kappa-B ligand, *RANKL*, and *CD276* molecule known as B7-H3. On the contrary, they had decreased transcript levels of the activating receptors *FCGR3A* (CD16), *KLRF1* (NKp80), or *KLRB1* (CD161) compared with resting and non-proliferating cells (Figure 4B). Furthermore, we noticed that transcriptional changes in proliferating NK cells extended also to differences in expression of HLA class I and II molecules. Remarkably, proliferating NK cells expressed high transcript levels of HLA class II, that correlates with their activated status, whereas transcripts corresponding to HLA class I molecules were decreased in comparison with resting and non-proliferating NK cells, representing an unexpected finding (Figure S5B in Supplementary Material). Altogether these results suggest the existence of differential activation responses of NK cells toward the stimuli provided by IAPs, which may translate into a rapid proliferation of certain subsets of NK cells compared with others with delayed or no proliferative response to the same stimuli.

### Phenotypic Hallmarks of Proliferating NK Cells

**Figure 5:**
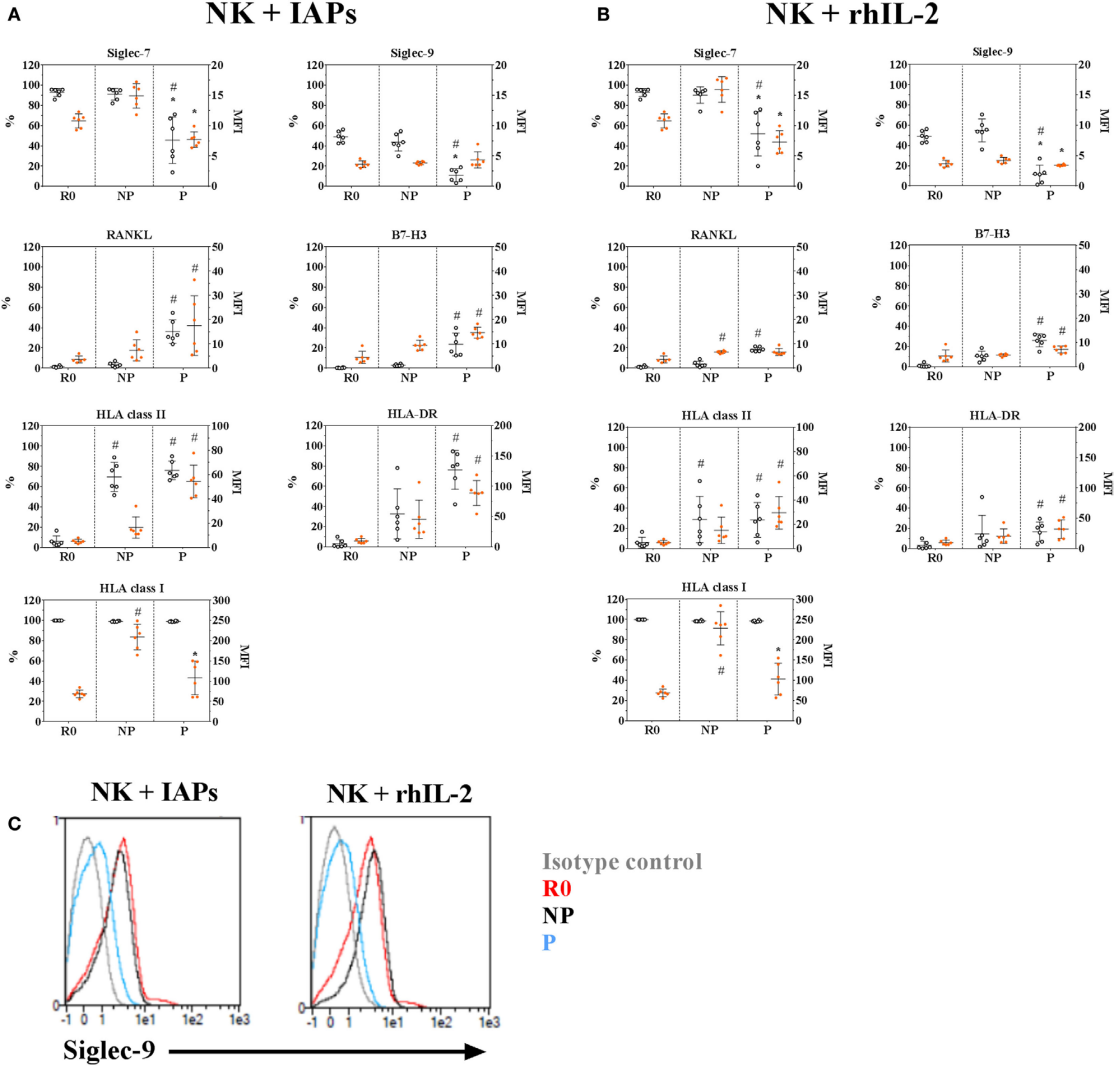
Phenotypic characteristics of proliferating and non-proliferating natural killer (NK) cells compared with resting NK cells. Surface expression of selected inhibitory, activating, and co-stimulatory receptors in 5-days proliferating (P) and non-proliferating (NP) NK cells compared with resting (R0) cells prior culture with IAPs **(A)** or with rhIL-2 only **(B)** were determined by flow cytometry (*n* = 6). Of note, values shown for HLA-DR correspond to a repeated analysis with six other donors. Results are represented for each marker as percentage of positive cells (white circles, referred to the left scale of the plots) and mean fluorescence intensity of positive cells (orange circles, referred to the right scale of the plots) showing mean values and SDs. Statistical analysis was performed using the Kruskal–Wallis test applying Dunn’s *post hoc* test. Only significant differences are shown as # for PvsR0 and * for PvsNP. **(C)** Representative histogram of Siglec-9 expression on NK cells at the different time points and proliferative status analyzed R0, P, or NP.

The changes observed in transcript levels of activating and inhibitory receptors and co-receptors are likely to extend at the protein level, and may reveal phenotypic characteristics of proliferating and non-proliferating NK cells. In addition, the detected phenotypic differences may depend on the stimuli used to promote *ex vivo* NK cell expansion. Therefore, we analyzed by flow cytometry, the expression of several selected molecules (Table S1 in Supplementary Material) in resting, proliferating, and non-proliferating NK cells expanded for 5 days with IAPs or rhIL-2 only. Most of the observed changes in protein content confirmed the results obtained by transcriptional profiling, with significant differences especially between resting and proliferating cells (Figure 5; Figure S6 in Supplementary Material). Among them, we identified that proliferating NK cells contained significantly decreased frequencies of cells expressing the inhibitory receptors Siglec-7 and -9, independently of the expansion protocol, compared with both non-proliferating and resting cells. Also, independently of the expansion method was a significant reduced expression of the activating receptors KLRB1, NKp80, CD16, and the inhibitory receptor KLRG1 within proliferating cells compared with resting cells (Figure S6 in Supplementary Material). All other analyzed activating receptors and co-receptors were expressed at higher levels in proliferating NK cells, except the NKG2D co-receptor 2B4. Despite almost all resting NK cells expressed 2B4 at the beginning of the culture, this could not be detected on day 5 of expansion with none of the expansion methods used (data not shown). This suggests an oscillating expression of 2B4 on NK cells during expansion, since expression of 2B4 has been reported after longer culture periods (23). Moreover, we could confirm RANKL and B7-H3 expression, absent in resting cells, appeared after 5 days of expansion. Their expression was mainly restricted to proliferating NK cells and reached higher levels when NK cells were expanded with IAPs instead of with rhIL-2 only (Figures 5A,B). Similarly, expression of HLA class II molecules and particularly HLA-DR, were increased after 5 days of expansion with IAPs compared with rhIL-2 only. Expression of HLA-DR persisted after longer term culture (data not shown). Interestingly, the reduction observed earlier in the expression of HLA class I molecules after 5 days of culture was verified here too, independently of the expansion protocol used.

### Co-Expression of HLA-DR, RANKL, and B7-H3 Define a Subset of Highly Proliferating NK Cells

**Figure 6:**
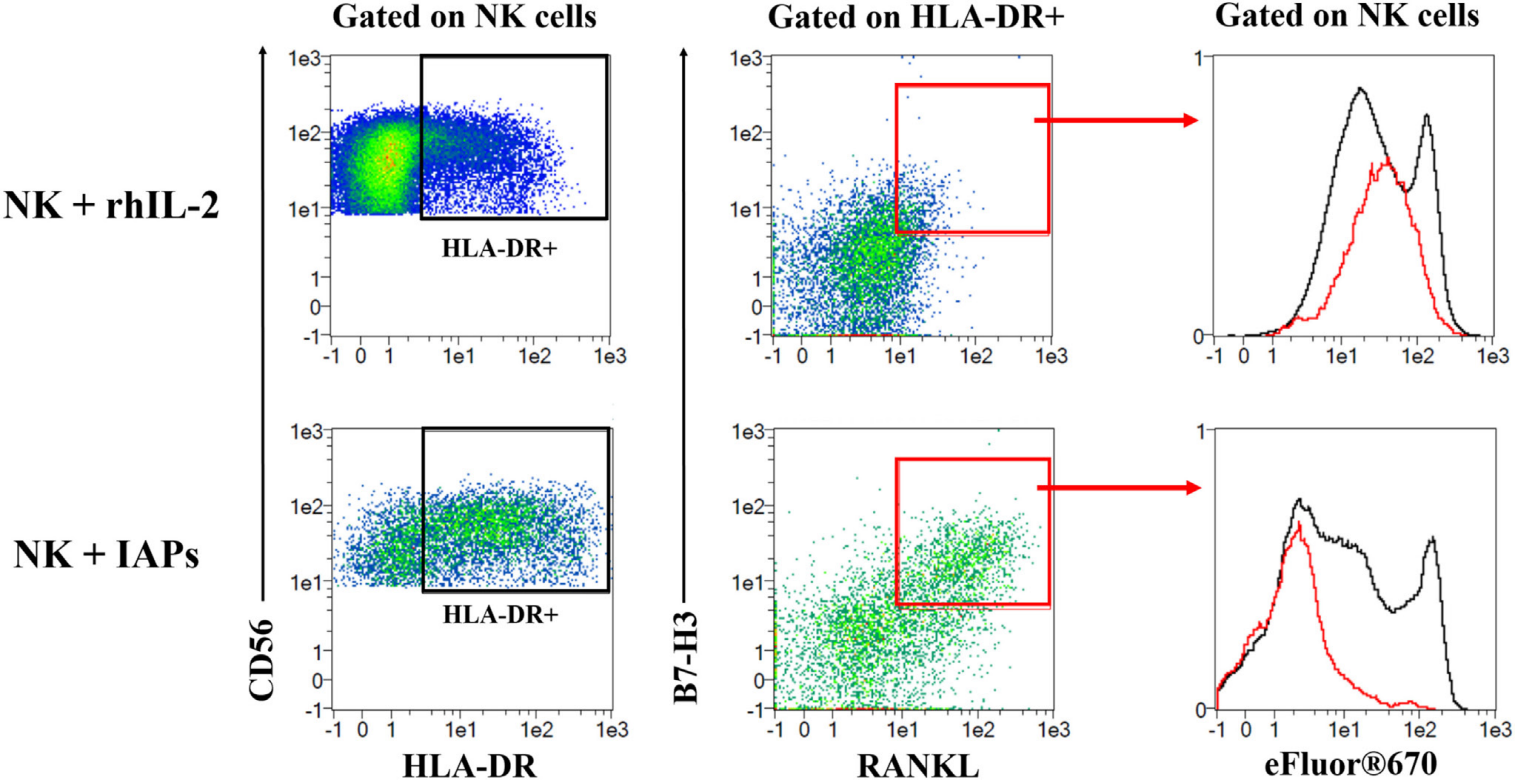
Identification of a subset of HLA-DR expressing natural killer (NK) cells co-expressing RANKL and B7-H3 with high proliferative capacity. eFluor^®^670 labeled NK cells were expanded in co-culture with IAPs or using rhIL-2 only for 5 days. Co-expression of HLA-DR, RANKL, and B7-H3 was analyzed at this time point by flow cytometry, and compared between the two different culture methods used. First, HLA-DR expressing NK cells were gated and used to assess expression of RANKL and B7-H3. A double positive RANKL and B7-H3 subpopulation of HLA-DR expressing cells was mainly detected in NK cells in co-culture rather than with rhIL-2 only. This subpopulation of HLA-DR^+^RANKL^+^B7-H3^+^ corresponded to the most highly proliferating as observed when depicted (red) against the total pool of expanded NK cells (black) based on the dilution of the eFluor^®^670 cell trace dye. Plots shown are representative of six different donors. The complete gating strategy is shown in Figure S7 in Supplementary Material.

Given that co-culture with IAPs for 5 days produced an enrichment of HLA-DR expressing cells and induced expression of RANKL and B7-H3, we investigated the correlation of the co-expression of these three molecules with the proliferative status of NK cells by flow cytometry. HLA-DR expressing NK cells contained a subpopulation of double positive RANKL and B7-H3 NK cells that corresponded to the most highly proliferating NK cells (Figure 6; Figure S7 in Supplementary Material). This was substantiated by NK cells co-cultured with IAPs in contrast to cultures with rhIL-2 only. The HLA-DR^+^RANKL^+^B7-H3^+^ NK cell population was minimally represented in cultures with rhIL-2 only, but enlarged in co-cultures with IAPs. In summary, we identified a subset of NK cells that preferentially expand in response to the stimuli provided by IAPs and is characterized by expression of HLA-DR, RANKL, and B7-H3.

## Discussion

Recent developments in *ex vivo* NK cell expansion protocols for clinical applications have exploited the use of autologous feeder cells in combination with cytokines to boost NK cell proliferation and their activation status (16–19). However, the components responsible for this enhancement remain somewhat elusive, and little is known regarding the phenotypic and intracellular changes occurring when NK cells start to actively proliferate under these conditions. In this study, we established a co-culture protocol using irradiated autologous CD56-depleted PBMCs (IAPs) as feeder cells, to use it as a tool for the analysis of factors influencing *ex vivo* proliferation of NK cells. After ascertaining the achievement of higher numbers of functional NK cells by co-culture with IAPs compared with standard culture with rhIL-2 only, we subsequently analyzed the influence of cell-to-cell interactions, soluble factors, and specific PBMCs subpopulations on NK cell proliferation. The need of homotypic and heterotypic cell-to-cell interactions for NK cell survival, activation, and proliferation has been previously reported (20, 31, 32). Consistent with this, disruption of cell-to-cell interactions between NK cells and IAPs by using Transwell^®^ plates results in lower NK cell proliferation than when feeder cells and NK cells are in close contact. Additionally, here we report that NK cell interactions with IAPs induce changes in production of soluble factors that improve NK cell *ex vivo* proliferation. The role of certain soluble factors, mostly of the cytokines IL-2, IL-15, and IL-21, in inducing mature human NK cell *ex vivo* proliferation has been well acknowledged (33, 34). In our set-up, sufficient IL-2 levels were achieved by supplementation of high concentrations of rhIL-2 at the beginning of the expansions, and also possibly by additional IL-2 production by T cells within the IAPs upon stimulation in culture. Concerning IL-15 and IL-21, we assessed their production using commercial enzyme-linked immunosorbent assays during a preliminary test on supernatants from 5-day expanded NK cells with and without IAPs. Levels of soluble IL-15 were undetected, and only background levels of IL-21 could be detected, without differences upon the presence or absence of IAPs. With this, we concluded that there was no significant production of neither IL-15 nor IL-21 in our co-culture system. Thus, our findings point to additional cytokines and other soluble factors produced during IAPs-NK cell co-cultures that should be further investigated, and may be used to improve *ex vivo* NK cell expansion methods without the use of feeder cells.

Considering the heterogeneous composition of IAPs, we analyzed the influence of subpopulations (mainly T cells, B cells, and monocytes) in promoting NK cell proliferation. In our co-culture method, the presence of both T cells and monocytes is sufficient to completely enhance *ex vivo* NK cell proliferation, whereas the presence of B cells is dispensable. We observed that monocyte depletion substantially reduces the production of cytokines from remaining feeder cells, which correlates with the lowest NK cell expansions. On the other hand, the presence of monocytes without T cells almost completely hindered cytokine production, although this caused a less prominent reduction of NK cell expansion. This indicates that the irradiated monocytes besides participating in promoting cytokine production from T cells, may also produce other soluble factors, or express certain surface molecules that result in promoting *ex vivo* NK cell proliferation. To a certain extent, this correlates with previous observations showing the potential of monocytes to boost *ex vivo* NK cell expansion (20, 35), but it is in contrast with the exclusion of a beneficial effect of T cells shown in one of the studies (20). The reason of this difference may be explained by the use of non-irradiated accessory cells and only rhIL-2 supplemented in this former study, while in our set-up the T cells are irradiated and OKT3 is additionally supplemented together rhIL-2. It is important to remark that full activation of peripheral blood T cells by OKT3 requires additional signals from accessory cells and the presence of IL-2 (36). These accessory cells are mainly monocytes that through their Fc receptors, bind OKT3, and induce crosslinking of the CD3 molecule on T cells leading to a proper stimulation (36–38). This may explain that upon the absence of monocytes, despite the presence of OKT3 and IL-2, cytokine production by T cells is severely hampered. Moreover, the effect of OKT3, murine IgG2a mAb, on NK cell activation through the Fc receptor CD16 would be minimal, as CD16 has no or low binding affinity to IgG2a isotype (37, 39). In summary, in our co-culture system, the interaction of irradiated T cells and monocytes through bidirectional binding of OKT3, together the presence of IL-2 seems sufficient to induce production of soluble factors at the beginning of the co-culture with clear further beneficial effects on NK cell *ex vivo* growth.

Several studies have performed transcriptional and microRNA expression analysis in human NK cells to determine global changes after short activation or long-term expansion (23, 40, 41). Using also whole genome expression analysis and functional annotation, we focused on transcriptional differences between proliferating NK cells and their remaining non-proliferating counterparts after 5-days in co-culture with IAPs. By choosing this time point for analysis, we assured the emergence of pro-survival and proliferation pathways, absent at earlier time points, or extinguished at later ones. Certainly, we identified among the most highly expressed transcripts in proliferating NK cells many molecules involved in regulation of cell cycle and mitosis. Interestingly also transcripts corresponding to the zinc finger proteins, *ZBED2* and *ZBTB32* were upregulated. While only little is known about ZBED2 (42), the transcription factor ZBTB32 recently has been described to play a role in controlling proliferation of mouse cytomegalovirus (MCMV)-specific NK cells (43). Since in that study ZBTB32 was predominantly involved in the context of infection and inflammation, but was not required for homeostatic proliferation of mouse MCMV-specific NK cells, it was surprising to find increased levels of a transcript corresponding to ZBTB32 in proliferating human NK cells in our co-culture system. Therefore, it will be interesting to analyze which role zinc finger proteins, particularly, ZBED2 and ZBTB32 play in modulating the *ex vivo* expansion of mature human NK cells.

We describe also several phenotypic characteristics of highly proliferating NK cells compared with non-proliferating and resting ones. Actively proliferating NK cells lose expression of the inhibitory receptors Siglec-7 and -9 compared with both resting and non-proliferating counterparts. Several studies have shown that blocking of Siglec-7 and -9 receptors enhances NK cell cytotoxicity against target tumors, allowing for a better activation (44, 45). The reduced expression of Siglec-7 and -9 in proliferating NK cells may reflect a differential activation status related to high proliferation. Also in this regard, we detected reduced expression of the activating receptors NKp80 and CD16 preferentially in the population of proliferating NK cells. Down-regulation of NKp80 and CD16 is known to occur early after NK cell activation (46–48). Therefore, decreased Siglec-7/-9, NKp80, and CD16 expression evidence that differential activation responses within the original pool of NK cells result in either active *ex vivo* proliferation or quiescence. Moreover, the expression of the molecules RANKL and B7-H3 was significantly induced in NK cells particularly proliferating in co-culture with IAPs, as opposed to cultures with rhIL-2 only. Although up-regulation of RANKL has been previously reported to occur in CD56^bright^ NK cells (49) little is known about its function in NK cells. On the other hand, B7-H3, a member of the B7/CD28 superfamily of costimulatory molecules is expressed in many human cancers and induced in several immune cells such as activated monocytes and dendritic cells with a controversial role in modulating T cell activity (50, 51). It would be interesting to determine whether B7-H3 is also a marker of activated NK cells *in vivo* and learn about its functional role.

Co-culture with IAPs also increased expression of particularly HLA-DR molecules in proliferating NK cells compared with culture with rhIL-2 only. Further analysis of the expression of RANKL and B7-H3 in HLA-DR expressing NK cells, revealed that a subpopulation of double positive RANKL and B7-H3 HLA-DR expressing NK cells, corresponds to the most actively expanded upon co-culture with IAPs. Increased frequencies of HLA-DR expressing NK cells have been observed already after few days of culture with IL-2, possibly due to clonal expansion of an original HLA-DR expressing NK cell subpopulation (52). Recently, the expression of HLA-DR molecules was described as a surrogate marker for NK cell clonality in chronic lymphoproliferative disorders (53). Our data support the hypothesis of a preferential clonal expansion of HLA-DR expressing NK cells possibly due to differential activation upon mitogens provided by IAPs as opposed to other NK cell subsets un-responsive to the same stimuli. Identification of the corresponding receptor(s) and pathway(s) responsive to pro-proliferative signaling will be key to optimize NK cell expansion protocols. Additionally, we describe a subset of HLA-DR^+^RANKL^+^B7-H3^+^ NK cells as the subset with the highest proliferative potential emerging under co-culture with IAPs. Subsequent studies may help to elucidate a functional role of RANKL and B7-H3 molecules in these highly proliferating NK cells. In addition, it will be interesting to verify the occurrence of a preferential expansion of HLA-DR expressing NK cells with other *ex vivo* expansion protocols, and whether this confers antigen presenting characteristics to NK cells, as it has been previously shown (54). Confirmation of antigen presenting properties of expanded NK cells may be further considered to extend the therapeutic potential of NK cells.

In summary, our findings uncover molecular and phenotypic characteristics of *ex vivo* proliferating NK cells and roles of autologous feeder cells that may have an impact in the development of new expansion methods and analysis strategies of expanded NK cells. In addition, we propose that characterization of the newly described subpopulation of HLA-DR^+^RANKL^+^B7-H3^+^ NK cells will be of interest for the further optimization of NK cell expansion protocols, and improved immunotherapeutic applications.

## Ethics Statement

This study was carried out using samples from healthy donors obtained from Klinikum Dortmund (Dortmund, Germany) with given consent from the Klinikum Dortmund to use those samples for research purposes.

## Author Contributions

MD-V designed and performed experiments, analyzed data, and wrote the manuscript; JK performed processing and statistical analysis of gene expression data and contributed to write the manuscript; UK provided advice and contributed to write the manuscript; and VH supervised research work, provided advice, and wrote the manuscript. All authors have discussed and revised the manuscript.

## Conflict of Interest Statement

The authors declare that the research was conducted in the absence of any commercial or financial relationships that could be construed as a potential conflict of interest.
