# Supplementary material for: Influence of Irradiated Peripheral Blood Mononuclear Cells on Both Ex Vivo Proliferation of Human Natural Killer Cells and Change in Cellular Property
Source: Front Immunol. 2017 Jul 24;8:854. doi: 10.3389/fimmu.2017.00854 (PMC5522833; doi:10.3389/fimmu.2017.00854)
Supplement: Supplementary file 1 [file data_sheet_1.docx]

Supplementary Material

Influence of Irradiated Peripheral Blook Mononuclear Cells on Both *Ex Vivo* Proliferation of Human Natural Killer Cells and Change in Cellular Property

María Delso-Vallejo^1*^, Jutta Kollet^1^, Ulrike Koehl^2^, Volker Huppert^1^

^1^Miltenyi Biotec GmbH, Bergisch-Gladbach, Germany

^2^Institute for Cellular Therapeutics, IFB-Tx, Hannover Medical School, Hannover, Germany

*** Correspondence:** María Delso-Vallejo [mar.delso15@gmail.com](mailto:mar.delso15@gmail.com)

# Supplementary Material

**RNA isolation amplification and labelling**

Total RNA was isolated using the NucleoSpin® RNA kit (Macherey-Nagel) and RNA quality and integrity were assessed with the Agilent 2100 Bioanalyzer platform (Agilent Technologies). The obtained RNA integrity numbers of all samples revealed values between 7.4 and 10, showing sufficient quality for gene expression profiling experiments according to published data (1). The RNA amount was quantified using the Qubit fluorometer (Invitrogen) and a maximum of 10ng of total RNA from each of the resting, proliferating and non-proliferating NK cell samples was amplified and labelled using the Agilent Low Input Quick Amp Labeling Kit (Agilent Technologies). Yields of complementary RNA (cRNA) and the dye (Cy3) incorporation rate were measured with the ND-1000 Spectrophotometer (NanoDrop Technologies).

**Hybridization, washing and scanning**

The hybridization procedure was performed according to the Agilent 60-mer oligo microarray processing protocol using the Agilent Gene Expression Hybridization kit (Agilent Technologies). Shortly, 600ng of Cy3-labeled fragmented cRNA in hybridization buffer was hybridized overnight (17h, 65°C) to Agilent Whole Human Genome Oligo Microarrays 8x60K V2 using Agilent´s recommended hybridization chamber and oven. Microarrays were washed once with the Agilent Gene Expression Wash Buffer 1 for 1min at room temperature and followed by a second wash with preheated (37°C) Agilent Gene Expression Wash Buffer 2 for 1 minute. Fluorescence signals of the hybridized Agilent Microarrays were detected using Agilents´s Microarray Scanner System (Agilent Technologies). The Agilent Feature Extraction Software (FES 10.7.3.1) was used to read out and process the microarray image files.

**Statistical analysis of microarray data**

Microarray data analysis was performed to compare gene expression profiles of freshly isolated (R0), 5-day proliferating (P), and 5-day non-proliferating (NP) NK cells co-cultured with IAPs from 5 different donors. First, normalized Log2 intensity values of all reporters with an annotated Gene ID (NCBI) were used to perform an unsupervised principal component analysis (PCA) by the R/SVD method with imputation using ClustVis (2). Furthermore, expression differences between groups of samples were evaluated using analysis of variance (ANOVA) test with repeated measurements design (‘donors’ in the error term) on normalized log2 intensity values of all reporters, followed by pairwise group comparisons with Tukey´s *post hoc* tests. The multiple testing problem was addressed by adjusting p-values using the Benjamini & Hochberg method. Reporters with annotated Gene IDs were filtered for statistical significance within the pairwise group comparisons (PvsR0, PvsNP and NPvsR0) according to the following selection criteria: ANOVA adjusted p-values < 0.05, Tukey p-values < 0.05 and median fold changes ≥ 2 or ≤ -2. All differentially expressed reporters of each of the pairwise comparisons were displayed in a Venn diagram using jvenn (3), and their corresponding median-centered log2 intensity values were hierarchically clustered (Euclidean distance, complete linkage method) and displayed in a heat map using the MeV software (TM4 suite, MeV_4_8_1) (4). Additionally, differentially expressed reporters were used to perform a functional annotation analysis (Miltenyi) to obtain enriched categories of biological processes and pathways for the differentially expressed genes. Differential signal intensities of reporters corresponding to activating, inhibitory, co-stimulatory receptors, HLA molecules and BTB-zinc finger transcription factors, were obtained for each donor by subtracting the log2-normalized signal intensity values of the reporters for each group comparison, and expressed as mean log2 ratios of signal intensities. The mean log2 ratios of signal intensities of differentially expressed reporters in proliferating compared to resting NK cells (PvsR0), was used to determine the 20 highest expressed transcripts in proliferating NK cells. The mean log2 values of signal intensities of these top 20 transcripts were displayed in a heat map for each group of samples (R0, NP, and P).

1. Fleige S, Pfaffl MW. RNA integrity and the effect on the real-time qRT-PCR performance. *Mol Aspects Med* (2006) **27**:126–139. doi:10.1016/j.mam.2005.12.003

2. Metsalu T, Vilo J. ClustVis: A web tool for visualizing clustering of multivariate data using Principal Component Analysis and heatmap. *Nucleic Acids Res* (2015) **43**:W566–W570. doi:10.1093/nar/gkv468

3. Bardou P, Mariette J, Escudié F, Djemiel C, Klopp C. jvenn: an interactive Venn diagram viewer. *BMC Bioinformatics* (2014) **15**:293. doi:10.1186/1471-2105-15-293

4. Saeed AI, Bhagabati NK, Braisted JC, Liang W, Sharov V, Howe EA, Li J, Thiagarajan M, White JA, Quackenbush J. [9] TM4 Microarray Software Suite. *Methods Enzymol* (2006) **411**:134–193. doi:10.1016/S0076-6879(06)11009-5

**Supplementary Figures and Tables**

**
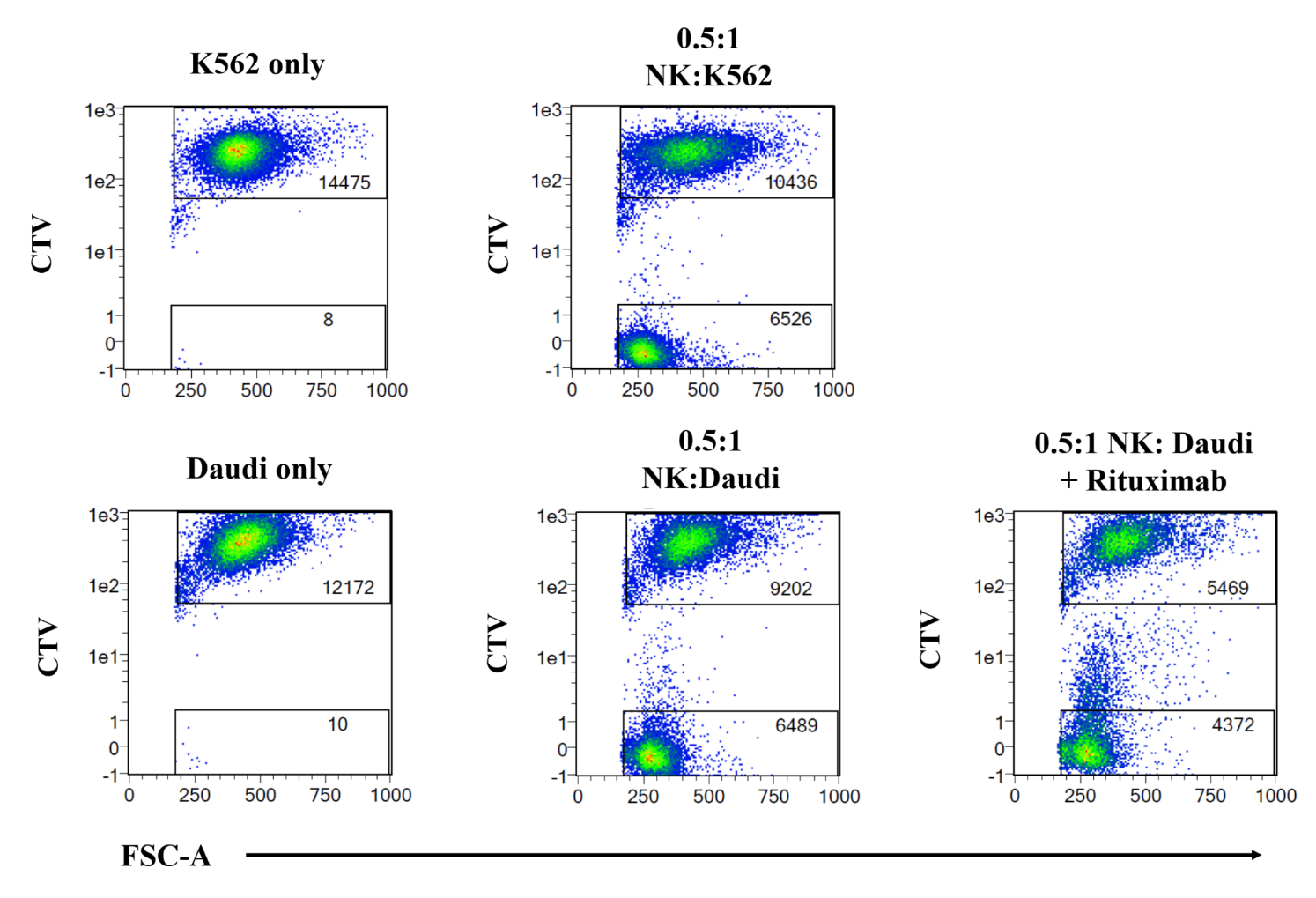
**

**Figure S1. Representative raw flow cytometry data of cytotoxicity assays.** Density plots corresponding to cytotoxicity assays using as effector cells NK cells expanded with IAPs, and as targets the tumor cell lines K562 and Daudi JP, corresponding to an effector-to-target ratio of 0.5:1. In the case of cytotoxicity against Daudi JP, 5 µg/mL of the anti-CD20 mAb rituximab were added to assess ADCC response from expanded NK cells. The CellTrace™ Violet Cell Proliferation dye (CTV) was used to label the target tumor cells (CTV^+^) to distinguish them from the effector NK cells (CTV^-^). Cells were gated on total viable cells, and numbers shown in the gates correspond to acquired cell counts. Counts of viable CTV^+^ cells in each E:T condition were used to calculate frequencies of specific cell lysis.


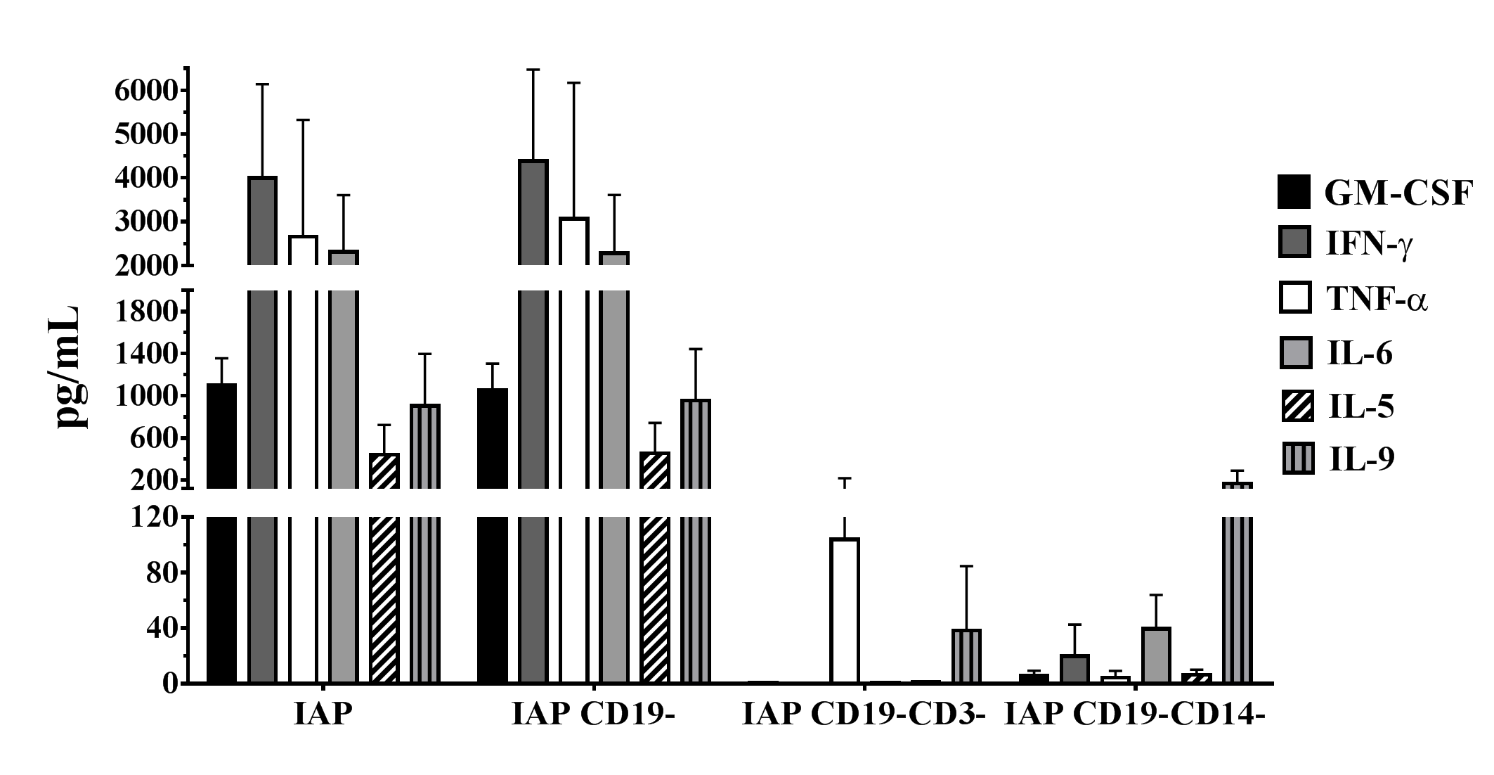


**Figure S2. Cytokine profiles of cell subsets-depleted feeder cell fractions cultivated without NK cells.** Different feeder cell fractions were cultured alone to serve as a control for the corresponding co-culture assay with autologous NK cells (see Figure 2E). Their potential to enhance ex vivo NK cell expansion was assessed by analysis of cytokine expression in the supernatant after 5 days of cultivation in complete medium by cytometric bead array (n=4). The following feeder cell fractions were investigated: depleted from CD56+ cells (IAPs), further depleted from CD19+ cells (IAP CD19-), and CD3+ cells (IAP CD19- CD3-), or CD14+ cells (IAP CD19-CD14-).


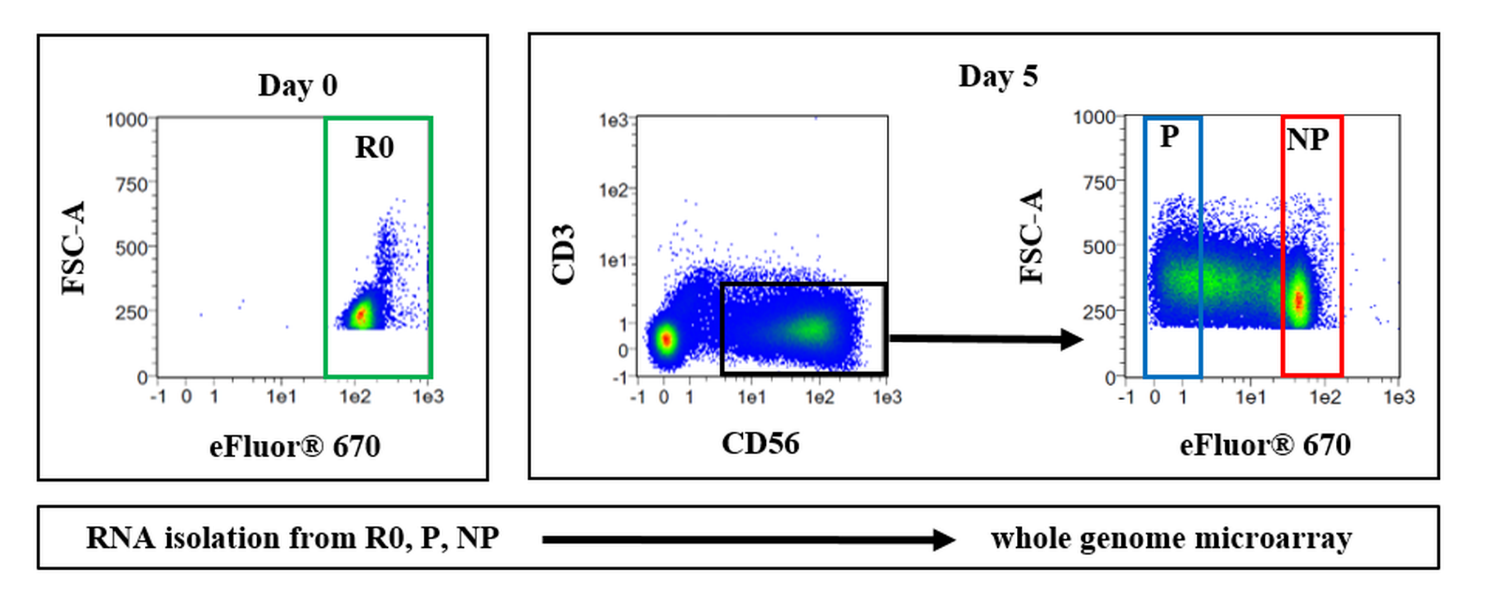


**Figure S3. Experimental design to obtain proliferating and non-proliferating NK cells after 5 days co-cultured with IAPs for further transcriptional and functional annotation analysis.** Workflow designed to obtain pure viable fractions of proliferating and non-proliferating NK cells to perform whole genome microarray analysis. NK cells were labelled with the cell division trace dye eFluo®670 prior cultivation with IAPs. After 5 days of culture, and prior selection of CD3^-^CD56^+^ NK cells, highly proliferating (P) cells corresponding to very diluted dye labelling, and non-proliferating (NP) corresponding to low diluted dye labelling, were sorted. The sorted fractions as well as respective samples collected on day 0 (R0) were lysed and RNA isolated for microarray analysis. R0, P and NP samples were collected from 5 different donors.

**
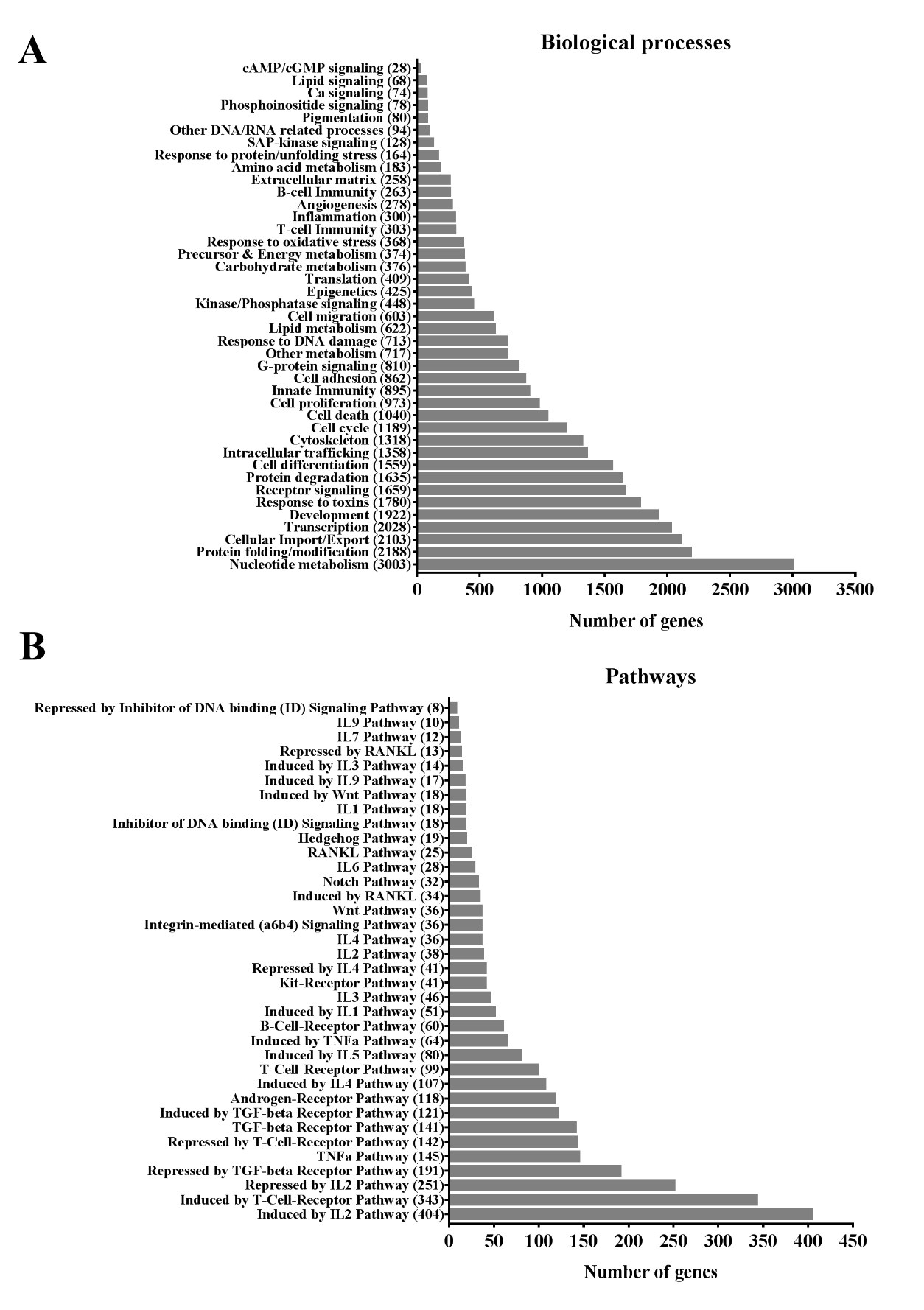
**

**Figure S4. Summary of biological processes and pathways associated with the differentially expressed transcripts among resting, proliferating and non-proliferating NK cells.** Results of the functional annotation analysis to determine which biological processes (A) and pathways (B) the 10,299 differentially expressed transcripts were related to. The number of candidate genes associated with each category is indicated in brackets.

**
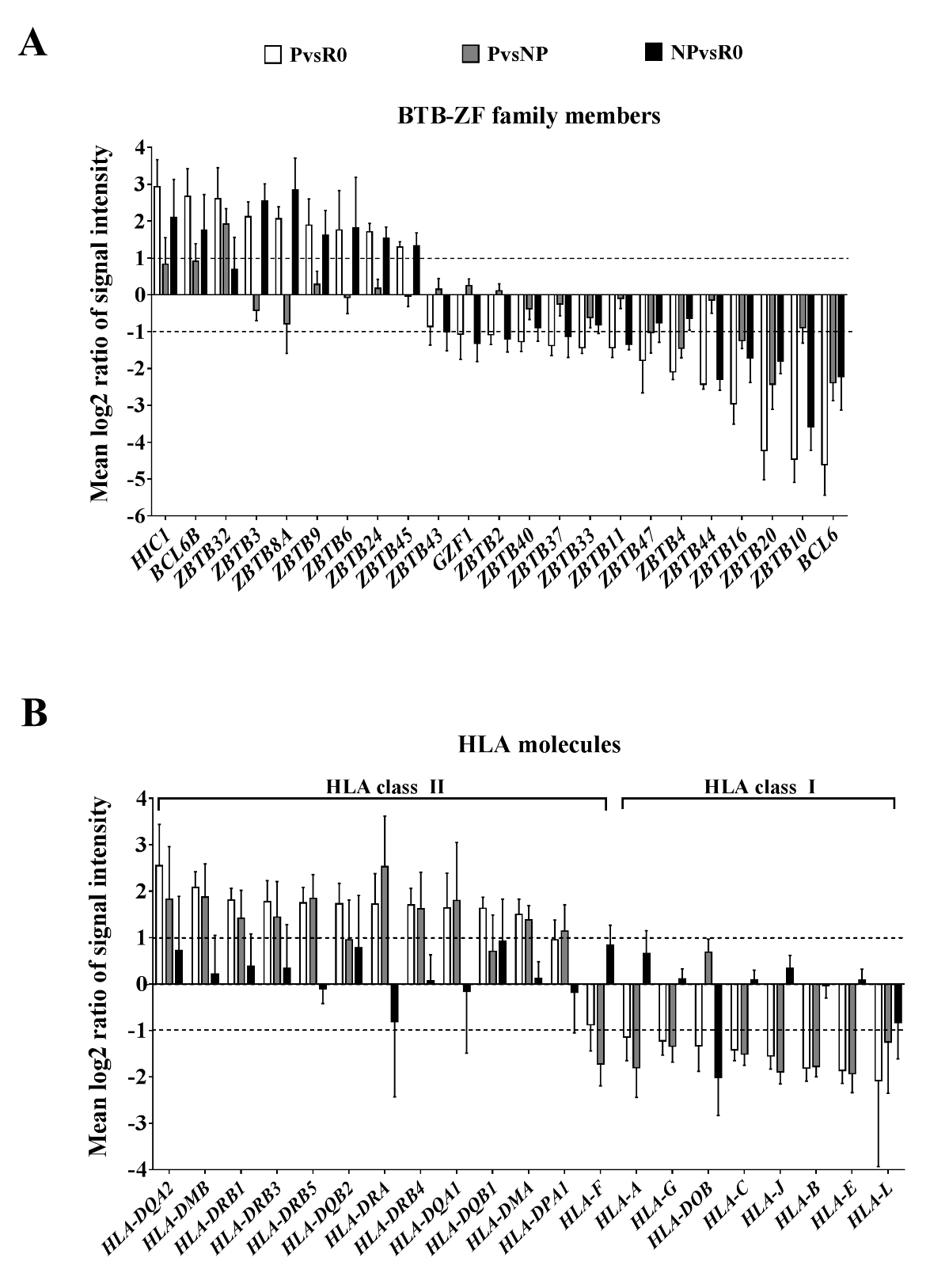
**

**Figure S5. Changes in transcript levels of members of the BTB-ZF family members and HLA-class I and II molecules among resting, proliferating and non-proliferating NK cells.** (A) Transcripts corresponding to BTB-ZF family members. (B) Transcripts corresponding to HLA molecules. Only transcripts with differential expression levels (mean log2 ratios ≥ 1 or ≤ -1, equivalent to ≥ 2 or ≤ -2 -fold difference) in at least one of the pair wise group comparisons are displayed. Data shown in all figures correspond to mean values with standard deviation of 5 different donors.


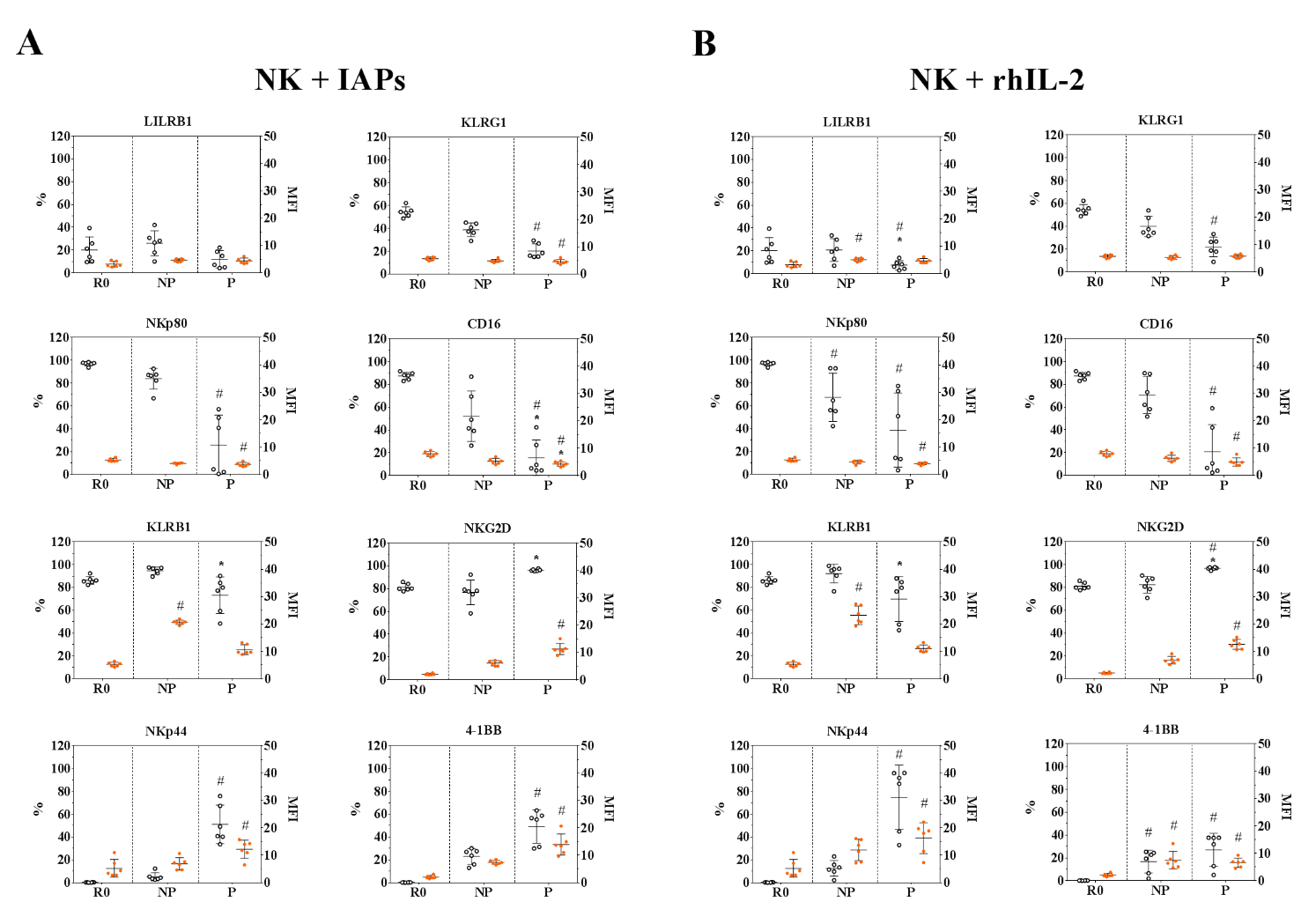


**Figure S6. Additional phenotypic characteristics of proliferating and non-proliferating NK cells compared to resting NK cells.** Surface expression pattern of selected inhibitory, activating and co-stimulatory receptors was assessed by flow cytometry in 5-days proliferating (P) and non-proliferating (NP) NK cells compared to resting (R0) cells prior cultivation with IAPs (A) or with rhIL-2 only (B) (n=6). Results are represented for each marker as percentage of positive cells (white circles, referred to the left scale of the plots) and mean fluorescence intensity (MFI) of positive cells (orange circles, referred to the right scale of the plots) showing mean values and standard deviations. Statistical analysis was performed using the Kruskal-Wallis test with Dunn´s *post hoc* test. Only significant differences are shown as # for PvsR0 and * for PvsNP.

**
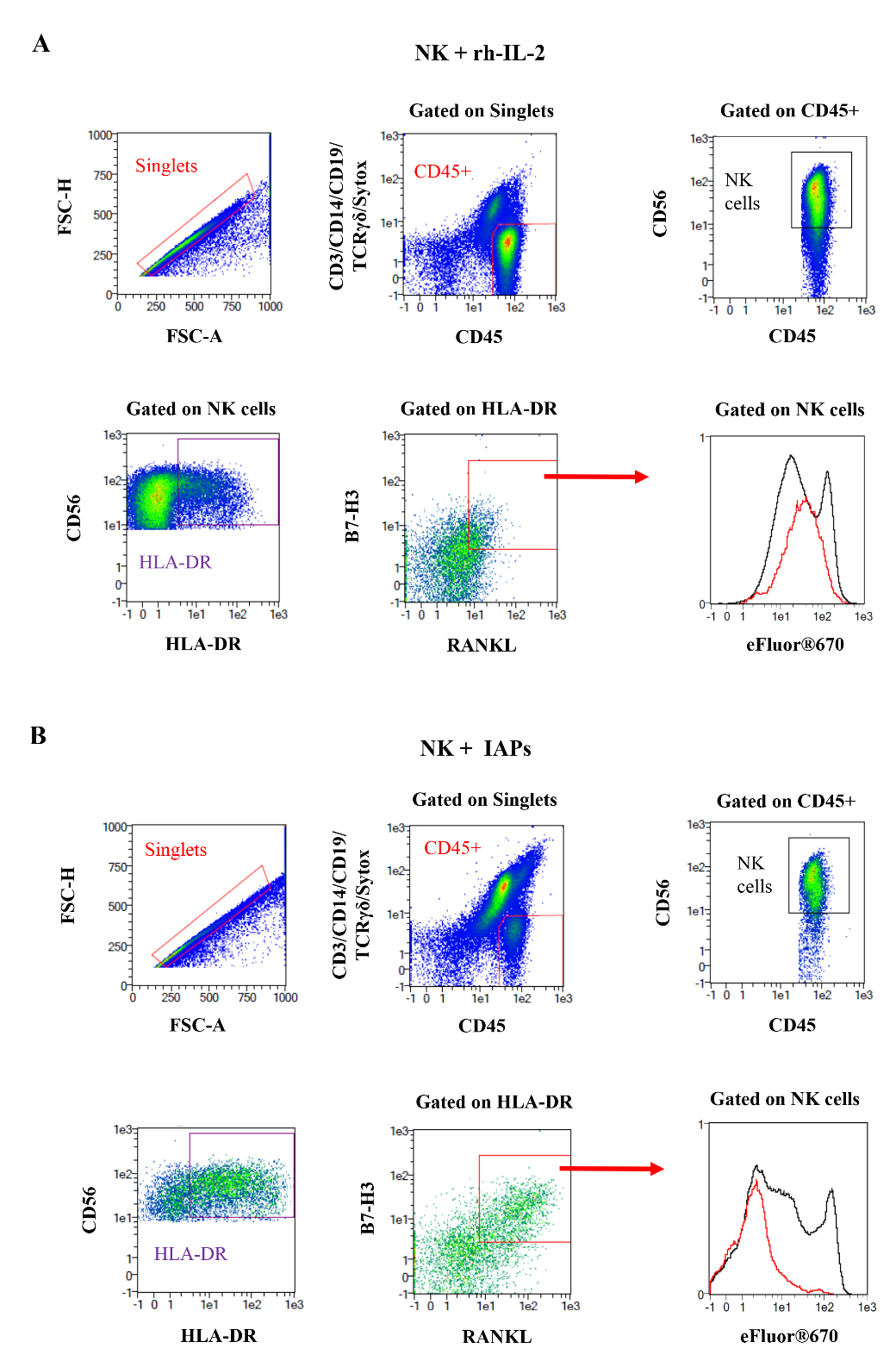
**

**Figure S7. Gating strategy to identify HLA-DR^+^RANKL^+^B7-H3^+^ highly proliferating NK cells.** NK cells were labeled with the cell trace dye eFluor®670 and analyzed after 5 days of culture with rhIL-2 only (A) or IAPs (B) for the expression of HLA-DR, RANKL and B7H3. Single cells were gated using forward side scatter height and area parameters and used to gate on CD45+ cells not expressing CD3, CD14, CD19, TCRγδ or SYTOX®Blue (dead cells). These CD45+ cells were plotted against CD56 to identify the population of NK cells. The gated NK cells were used to detect expression of HLA-DR. Subsequently HLA-DR+ NK cells were gated to analyse the expression of both RANKL and B7H3. Double positive RANKL and B7H3 HLA-DR expressing NK cells were gated and displayed (red) in a plot showing eFluor®670 dilution of total gated NK cells. Plots correspond to one donor as an example of 6 different donors analyzed.


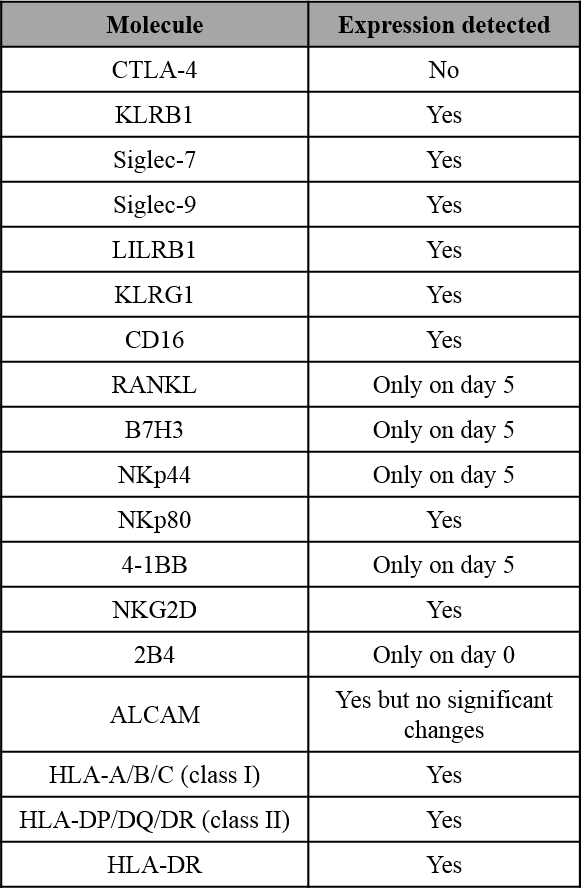


**Supplementary table 1. Expression of surface molecules analyzed in resting (R0), proliferating (P) and non-proliferating (NP) NK cells prior and after 5 days of culture with IAPs or rhIL-2 only.** The expression of nearly all molecules analysed was detected by flow cytometry and showed significant changes (p-values < 0.05) among the groups. Only CTLA-4, expression could not be detected at any of the time points, and ALCAM expression was not significantly changed among the groups.
